# Programmed death-ligand 1 expression in rectal cancer

**DOI:** 10.1007/s10353-016-0447-8

**Published:** 2016-10-07

**Authors:** G. Jomrich, G. R. Silberhumer, B. Marian, A. Beer, L. Müllauer

**Affiliations:** 1 Department of Surgery, Medical University of Vienna, Spitalgasse 23, 1090 Vienna, Austria; 2 Institute of Cancer Research, Medical University Vienna, Borschkegasse 8a, 1090 Vienna, Austria; 3 Clinical Institute of Pathology, Medical University Vienna, Währinger Gürtel 18–20, 1090 Vienna, Austria

**Keywords:** Rectal cancer, Programmed death ligand 1, Tumor-infiltrating lymphocytes, Neoadjuvant chemoradiation, Total mesorectal excision

## Abstract

**Background:**

Colorectal cancer (CRC) is the fourth most common cause of death worldwide. Approximately 30 % of all CRC occurs in the rectum. Improvements in survival rates were achieved thanks to multimodal therapy, combining surgery and chemoradiation. Nevertheless, the prognosis of patients suffering from rectal cancer (RC) remains poor. Programmed cell death protein 1 (PD-1) and its ligand programmed death ligand 1 (PD-L1) regulate tumor immune response. The aim of this study was to analyze the expression of PD-L1 in RC pre- and post-neoadjuvant therapy and evaluate PD-L1 as a biomarker and potential target for therapy.

**Methods:**

In all, 29 patients with RC treated at the Medical University Vienna who received preoperative chemoradiation were retrospectively enrolled in this study. Expression of PD-L1 was investigated by immunohistochemistry with two different anti-PD-L1 antibodies.

**Results:**

No PD-L1 expression on cancer cells could be observed in all 29 cases in the specimens before chemoradiation as well as in the surgical specimens after neoadjuvant therapy. In one of the two staining methods performed, five (17.24 %) post-chemoradiation cases showed faint lymphohistiocytic staining.

**Conclusion:**

No expression of PD-L1 in RC cells before and after chemoradiation was found in our collective of 29 patients. Further investigations to evaluate the role of PD-L1 as a potential therapeutic target in RC are urgently needed.

## Introduction

Colorectal cancer (CRC) is one of the leading causes of cancer-related death worldwide [1]. The standard treatment of locally advanced rectal cancer (RC) is neoadjuvant chemoradiation (NCR) followed by surgical resection [2]. Despite the usage of multimodal neoadjuvant therapy and improved survival rates, the prognosis of patients with RC remains unsatisfying. After neoadjuvant treatment combined with surgical resection, histopathological response rates differ enormously [3]. In recent years, significant insights into the interactions between the immune system and cancer cells have been gained. Recently, programmed death ligand 1 (PD-L1) raised scientific interest, as the first clinical studies with PD-L1 inhibitors promised encouraging results in several tumor types [4–7].

The binding of PD-L1 to its receptor programmed cell death protein 1 (PD-1) plays a major role in the interaction between cancer and the immune system. PD-1 belongs to the CD28/CTLA-4 immunoglobulin family and is expressed on activated T and B cells, monocytes, and tumor-infiltrating lymphocytes (TILs). PD-L1 can be found on resting T cells, B cells, macrophages, and vascular endothelial cells [8, 9]. The PD-1–PD-L1 interaction plays a key role in maintaining self-tolerance to protect against severe self-damage while the immune system is activated because of infections. Tumor cells can overexpress PD-L1, which binds to PD-1 on T cells and inhibits their activation. As a result, the tumor escapes surveillance by the immune system [10–12].

PD-L1 has been reported to be expressed in a number of malignancies, especially by glioblastoma, non-small-cell lung cancer, melanoma, renal cell carcinoma, and esophageal cancer [13–17]. Although PD-L1 is present in the cytoplasm and on the plasma membrane of tumor cells, not every type of cancer expresses PD-L1 [8, 18]. Clinical trials report promising data, when investigating PD-1 and/or PD-L1 blockade using monoclonal antibodies. Nevertheless, only a small number of patients benefit from inhibiting PD-1 or PD-L1 [7, 19, 20].

Recently, a difference in expression of PD-L1 in post-neoadjuvant therapy tumor tissue compared with pre-neoadjuvant therapy tumor tissue was found for various cancers [21–23]. To date, the expression of PD-L1 in neoadjuvant-treated RC has not been investigated intensively. In this single-center study, we examined the effect of neoadjuvant treatment on the expression of PD-L1 of locally advanced RC.

## Material and methods

### Patients

**Fig. 1:**
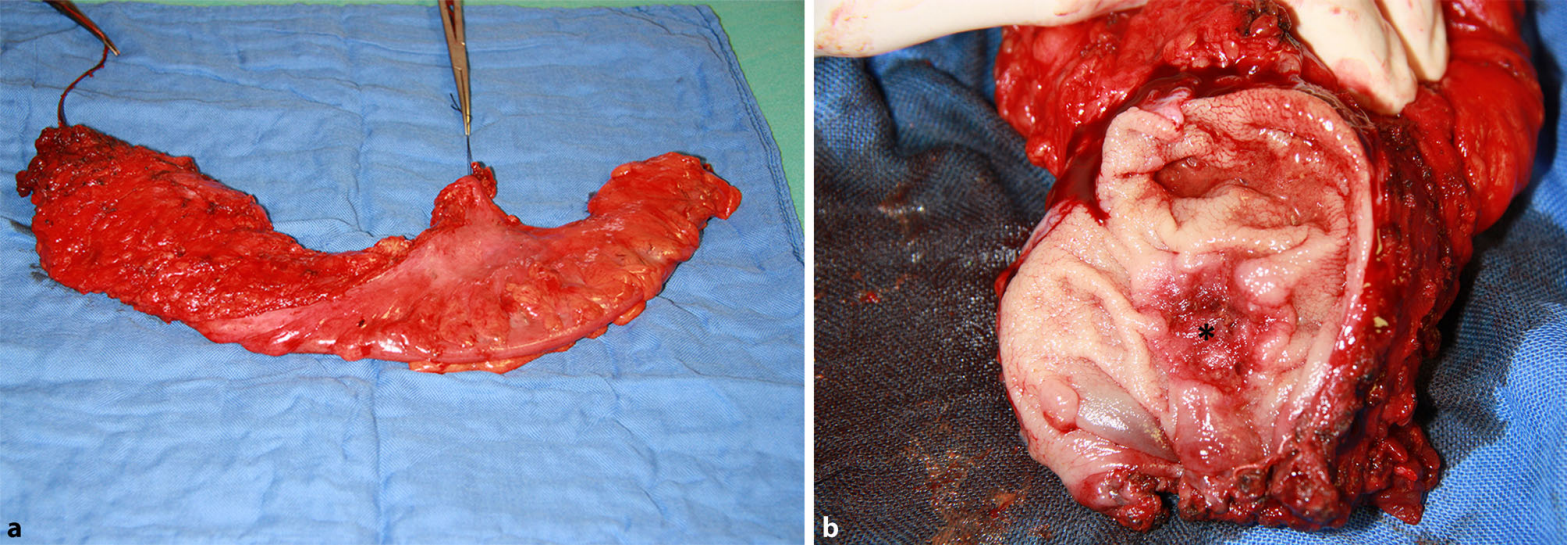
**a** Total mesorectal excision specimen. **b** * Rectal cancer after neoadjuvant chemoradiation showing areas of fibrosis indicating good response

In this retrospective analysis all patients underwent surgery for RC at the Department of Surgery, Medical University of Vienna, between 1 January 2012 and 31 December 2013. The study was approved by the Ethics Committee of the Medical University of Vienna, Austria, according to the Declaration of Helsinki. The study population consisted of patients who underwent total mesorectal excision (TME) after receiving capecitabine-based neoadjuvant chemoradiation (NCR) at the University Hospital Vienna, Austria (Fig. 1). Chemoradiation was delivered as long-course radiation (LCRT) with 50.4 Gy (1.8 Gy in 28 fractions). Patients presenting with distant metastatic disease at the time of diagnosis were excluded from the analysis.

PD-L1 expression in the context of NCR was investigated on pre-NCR biopsies and post-NCR surgical specimens. The clinicopathological factors selected and analyzed were age, gender, TNM staging according to the Union Contre le Cancer (UICC) 7th edition TNM classification, and the response rate to NCR [24].

### Assessment of response to NCR

Post-NCR specimens were analyzed for tumor response to neoadjuvant treatment. Response to neoadjuvant chemoradiation is reported as the ratio of viable tumor tissue and fibrosis. Tumor response rates were analyzed according to Dworak et al. [25]. Response was graded as: 0 – no regression; 1 – dominant tumor mass with obvious fibrosis and/or vasculopathy; 2 – dominantly fibrotic changes with few tumor cells or groups; 3 – very few (difficult to find microscopically) tumor cells in fibrotic tissue with or without mucous substance; 4 – no tumor cells, only fibrotic mass (total regression or response).

### Immunohistochemistry

Immunohistochemistry (IHC) was performed on paraffin-embedded specimens fixed in 4 % buffered formalin, using 3‑µm-thick histological sections. For PD-L1 expression in pre- and post-NCR tissue, two different staining methods were used. The first staining method was performed on a Ventana® BenchMark ULTRA (Roche, Tucson, AZ), automated IHC slide staining system, using the universal DAB detection kit with cell conditioning 1 (CC1) buffer of pH 8 and the PD-L1 (E1L3N®) XP® rabbit mAb (Cell Signalling Technology, Danvers, MA).

The second staining method was performed manually, using the antibody 5H1 (monoclonal antibody of human B7H1, Dr. Lieping Chen’s lab, isotype: mouse IGg1) as described previously [18].

PD-L1 expression was scored as positive or negative with semiquantitative estimation of percentage of positive tumor and stroma cells. Slides stained according to the first staining method were independently reviewed and scored by two observers (M.L. and J.G.). All slides stained according to the second staining method were reviewed and scored by M.B.

### Statistical analysis

Correlations between clinicopathological factors and expression of PD-L1 were analyzed with the χ^2^ test or Fisher’s exact test. Statistical analyses were performed, using SPSS 21.0 (IBM Corp.^©^, SPSS^©^) software.

## Results

The final study population consisted of 29 patients (82.76 % males and 17.24 % females), who were eligible for further investigations. Overall median age at the time of surgery was 65 years (23–85 years). The majority of patients (68.97 %) were radiologically in advanced UICC stages at the time of diagnosis (Table 1).

**Table 1:** Clinicopathological characteristics and response rate to NCR

Clinicopathological characteristics					
Factor					
*Age (years) n* =29					
Median age	65	(23–85)	–	–	–
Male	24	(82.76)	–	–	–
Female	5	(17.24)	–	–	–
–	pre-NCR (*n* = 29)			post-NCR (*n* = 29)	
*Tumor grading (%)*					
G2	24	(82.76)	G2	17	(58.63)
G3	5	(17.24)	G3	7	(24.13)
Gx	0	(0)	Gx	5	(17.24)
*Tumor stage (%)*					
cT0	0	(0)	ypT0	3	(10.35)
cT1	0	(0)	ypT1	2	(6.90)
cT2	1	(3.45)	ypT2	8	(27.57)
cT3	22	(75.86)	ypT3	14	(48.28)
cT4	6	(20.69)	ypT4	2	(6.90)
*Lymph node status (%)*					
cN0	9	(31.03)	ypN0	19	–
cN1	6	(20.69)	ypN1	7	(24.13)
cN2	14	(48.28)	ypN2	3	(10.35)
*UICC stage (%)*					
0	0	(0)	–	3	(10.35)
I	1	(3.45)	–	9	(31.03)
II a	7	(24.13)	–	7	(24.13)
II b	1	(3.45)	–	0	(0)
III a	0	(0)	–	1	(3.45)
III b	6	(20.69)	–	6	(20.69)
III c	14	(48.28)	–	3	10,35
*Response rate* ^a^ * to NCR (%)*					
4	–	–	–	3	(10.35)
3	–	–	–	15	(51.72)
2	–	–	–	4	(13.79)
1	–	–	–	3	(10.35)
0	–	–	–	4	(13.79)

*NCR* neoadjuvant chemoradiation, *UICC* Union Contre le Cancer

^a^According to Dworak et al.

All study patients received LCRT and completed NCR.

The histopathological results of the surgical specimens showed three patients (10.34 %) with complete response (CR, grade 4), 22 patients (75.86 %) with partial response (PR, grades 3–1), and four patients (13.8 %) with no response to NCR at all (nonresponders, NR; details in Table 1).

**Fig. 2:**
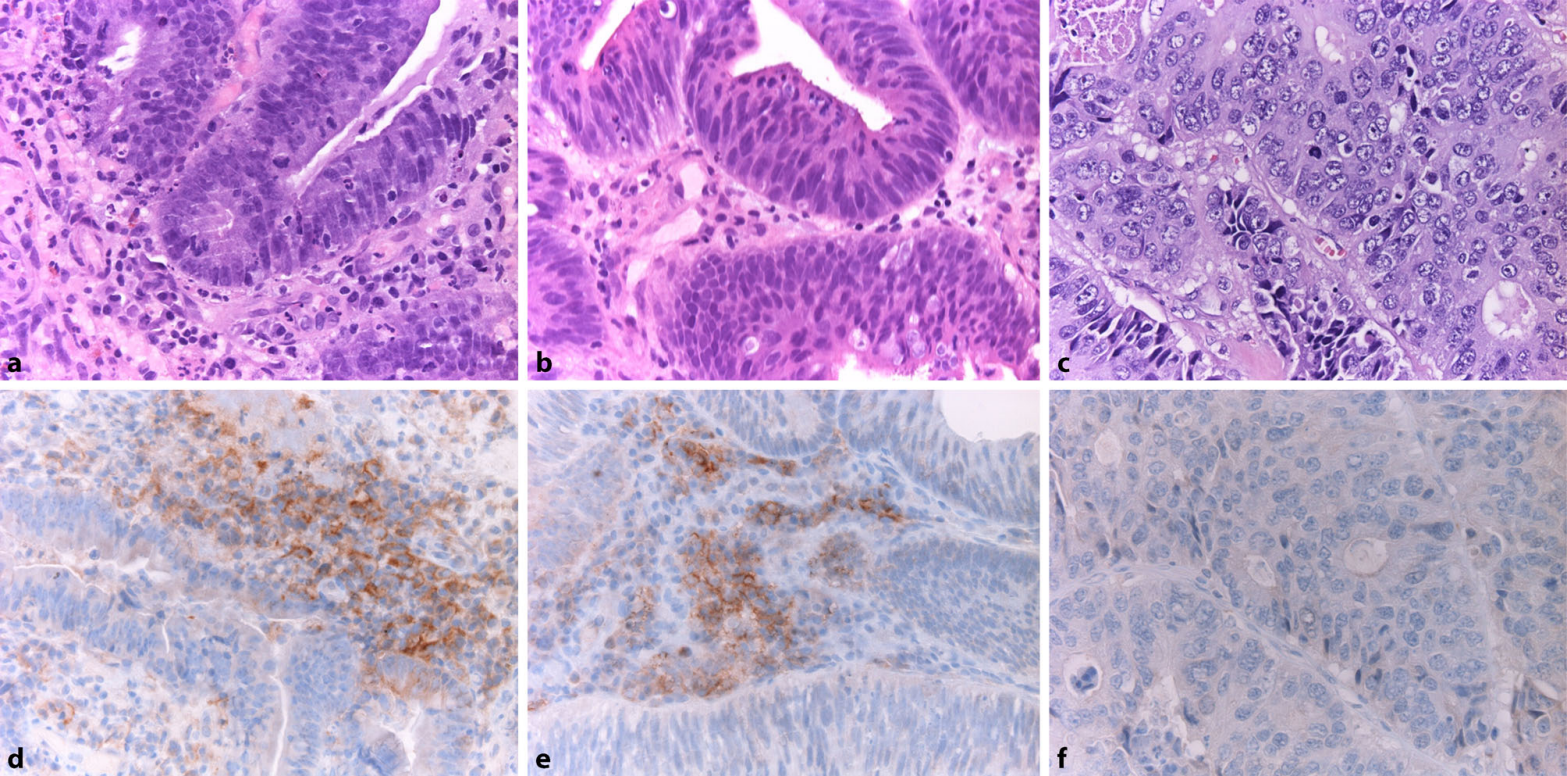
**a–c** Representative hematoxylin–eosin (H&E)-stained rectal cancer (RC) specimens after neoadjuvant chemoradiation (NCR) (magnification ×400). **d,** **e** Corresponding immunohistochemical PD-L1 stainings, showing PD-L1-positive immune cells in the tumor stroma of RC after NCR (magnification ×400). **f** RC showing no expression of PD-L1 in immunohistochemical PD-L1 staining after NCR (magnification ×400)

For both immunohistochemical staining methods employed, no expression of PD-L1 on neoplastic cells could be detected in all pre-NCR (29 cases, 100 %), as well as in all post-NCR cases (29 cases, 100 %). In five post-NCR cases (17.24 %) stained according to the first staining method, faint lymphohistiocytic staining (intensity score 1) of TILs could be observed (Fig. 2). Two of those five cases showed CR, two PR, and one was NR (Table 2).

**Table 2:** Pre-and post-NCR expression of PD-L1 in all 29 cases, using two different antibodies for staining

Expression of PD-L1 before and after NCR				
*Factor*	*Antibody E1L3N*		*Antibody 5H1*	
	(*n* = 29)	(%)	(*n* = 29)	(%)
*PD-L1 expression tumor pre-NCR*				
0	29	(100)	29	(100)
1	0	(0)	0	(0)
2	0	(0)	0	(0)
3	0	(0)	0	(0)
*PD-L1 expression stroma pre-NCR*				
0	29	(100)	29	(100)
1	0	(0)	0	(0)
2	0	(0)	0	(0)
3	0	(0)	0	(0)
*PD-L1 expression tumor post-NCR*				
0	29	(100)	29	(100)
1	0	(0)	0	(0)
2	0	(0)	0	(0)
3	0	(0)	0	(0)
*PD-L1 expression stroma post-NCR*				
0	24	(82.76)	29	(100)
1	5	(17.24)	0	(0)
2	0	(0)	0	(0)
3	0	(0)	0	(0)

*NCR* neoadjuvant chemoradiation

## Discussion

Even after adequate surgery, still around 30 % of patients with RC suffer from metastatic disease within 5 years of surgery. Currently established adjuvant chemotherapeutic agents have limited potential to prevent recurrence [2, 3]. Alternative treatment options are currently tested in clinical trials to improve long-term outcome [26].

Immunotherapy offers new opportunities in cancer treatment. Anti-PD-L1 antibodies showed encouraging results in different cancer patients, especially melanoma and recently non-small-lung cancer [19, 20]. In comparison with standard treatment, using chemo- and/or radiotherapy, immunotherapy is relatively safe, effective, and has low-grade side effects. Consecutively, this treatment modality gained high interest in CRC, too [27, 28].

Recently, Droeser et al. analyzed nearly 1,500 CRC samples, showing more than 30 % PD-L1 expression in untreated tumor tissue [29]. In contrast to other cancer types [21, 22], limited data are published on PD-L1 expression in RC and a potential impact of neoadjuvant therapy on PD-L1 expression. Therefore, the aim of this study was to investigate the expression of PD-L1 in a cohort of 29 neoadjuvant treated-patients suffering from locally advanced CRC.

Unfortunately, we could not detect PD-L1 expression neither in pre-NCR nor in post-NCR tumor tissues. Nevertheless, we observed expression of PD-L1 on TILs, which are considered to represent the immune response of the host to a malignant tumor, in five post-NCR cases. Expression of PD-L1 in 17.24 % of post-NCR TILs might implicate that the PD-L1/TAM (tumor-associated macrophages) and PD-1/TIL pathway is activated.

The significantly higher rate of PD-L1 expression in the study by Droeser et al. might be explained by a different study population combining patients with colon cancer and RC. We know from Xiao and Freedman that a microsatellite instable (MSI) subset of CRC might be a promising target for checkpoint immunotherapy [30]. Although Devaraj et al. reported that MSI in RC is rare, to our knowledge, little is known about the expression of PD-L1 in an MSI subset of RC to date [31].

Therefore, even though Droeser et al. reported more than 30 % PD-L1 expression in CRC tumor tissue, further investigations in developing PD-L1/PD-1 based immunotherapy should focus on the cell-type-specific analysis of the tumor surrounding the stroma. As mentioned, an MSI subset of CRC might be a promising target for checkpoint immunotherapy [30]. Based on these results, the MSI subset might be a potential therapeutic target in RC as well. Therefore, the role of PD-L1 as biomarker or target in immunotherapy in neoadjuvant-treated RC needs further investigation.
